# Serum sTREM2: A Potential Biomarker for Mild Cognitive Impairment in Patients With Obstructive Sleep Apnea

**DOI:** 10.3389/fnagi.2022.843828

**Published:** 2022-05-09

**Authors:** Xu Jiahuan, Zou Ying, Jin Hongyu, Wei Zhijing, Guan Shibo, Deng Chengyue, Fu Liangyu, Liu Fan, Wang Wei

**Affiliations:** ^1^Department of Respiratory and Critical Care Medicine, The First Hospital of China Medical University, Shenyang, China; ^2^Department of Rehabilitation Medicine Center, Shengjing Hospital Affiliated to China Medical University, Shenyang, China

**Keywords:** obstructive sleep apnea, mild cognitive impairment, soluble triggering receptor expressed on myeloid cells 2, serum, biomarker

## Abstract

**Objective:**

Cognitive impairment is a common comorbidity in patients with obstructive sleep apnea (OSA) that leads to poor quality of life and a heavier medical burden. However, the assessment and longitudinal tracking of cognitive impairment in OSA is challenging. This study aimed to examine the alternation and related factors of serum soluble triggering receptor expressed on myeloid cells 2 (sTREM2) in patients with OSA, and to explore whether serum sTREM2 could be a biomarker for mild cognitive impairment in OSA patients.

**Methods:**

A total of 94 OSA patients and 13 snoring subjects were enrolled in this cross-sectional study. Demographic information, questionnaires, and polysomnography results were collected. Serum sTREM2 levels were quantified using an enzyme-linked immunosorbent assay. Multivariate linear regression was used to analyze the factors influencing sTREM2, and the receiver operating characteristic curve was used to assess the predictive value of serum sTREM2 for mild cognitive impairment in patients with OSA.

**Results:**

Patients with OSA had higher serum sTREM2 levels than the controls. Multivariate linear regression analysis showed that serum sTREM2 levels in patients with OSA were associated with the Montreal Cognitive Assessment score and oxygen depletion index levels. Additionally, serum sTREM2 levels were higher in OSA patients with mild cognitive impairment (MCI) than in those without. The receiver operating characteristic curve showed that at a cutoff value of >18,437 pg/ml, the sensitivity of serum sTREM2 to predict MCI in OSA was 64.62%, the specificity was 68.97%, and the area under the curve was 0.70 (95% CI: 0.58–0.81).

**Conclusion:**

Serum sTREM2 levels were elevated in patients with OSA, particularly in those with MCI. It therefore has the potential to be a biomarker for MCI in OSA patients.

## Introduction

Obstructive sleep apnea (OSA) is the most common sleep-breathing disorder that occurs in 14% of men and 5% of women (Peppard et al., 2013). Owing to the intermittent hypoxia and sleep fragmentation, the main pathophysiological characteristics of OSA are associated with many complications, such as cancer, metabolic diseases, cardiovascular diseases, neurodegeneration diseases (Owen et al., 2021; Pelaia et al., 2021; Schernhammer et al., 2021; Wang et al., 2021). It has been reported that OSA is an independent risk factor for the development of cognitive impairment manifested as a decline in attention, long-term memory, executive function, visuospatial ability, and information processing (Leng et al., 2017; Zhu and Zhao, 2018). Cognitive impairment plays a role in reduced quality of life, increased healthcare costs, and increased mortality (Li et al., 2021; Xiong et al., 2021). However, the assessment and longitudinal tracking of cognitive impairment in OSA is challenging.

Triggering receptor expressed on myeloid cells 2 (TREM2) is a type I transmembrane protein mainly expressed in microglia in the central nervous system, which influences microglial activation, survival, and phagocytosis (Ulland and Colonna, 2018). Variants in TREM2 have been described as risk factors for Alzheimer’s disease (Guerreiro et al., 2013). TREM2 releases its extracellular segment as a soluble form (sTREM2) into the extracellular space, which can be detected in the cerebrospinal fluid (CSF) and peripheral serum (Wunderlich et al., 2013). It has been considered that the level of CSF sTREM2 is increased in patients with Alzheimer’s, and Parkinson’s diseases, even before symptom onset (Suárez-Calvet et al., 2016a; Mo et al., 2021; Morenas-Rodríguez et al., 2022). Recently, studies have found a significant association between increased serum/plasma sTREM2 levels and cognitive impairment in the elderly population, non-obese patients with diabetes, and young adults with Down syndrome (Ohara et al., 2019; Tanaka et al., 2019; Weber et al., 2020). Both CSF and serum/plasma sTREM2 are ideal biomarkers for these diseases.

Further studies showed that the level of sTREM2 in the CSF was positively correlated with the Pittsburgh Sleep Quality Index (PSQI) score (Hu et al., 2021), and that sTREM2 in the plasma was correlated with peripheral inflammatory cytokines (Weber et al., 2020). As sleep disturbance and higher inflammatory factors result from oxidative stress in OSA patients, we hypothesized that sTREM2 levels may be elevated in OSA patients and may be a potential biomarker for cognitive impairment in OSA. However, there is relatively little information regarding the role of sTREM2 in patients with OSA. Therefore, this cross-sectional study aimed to evaluate alterations in serum sTREM2 in OSA patients and explore the relationship between serum sTREM2 and cognitive impairment in OSA.

## Materials and Methods

### Subjects

**FIGURE 1:**
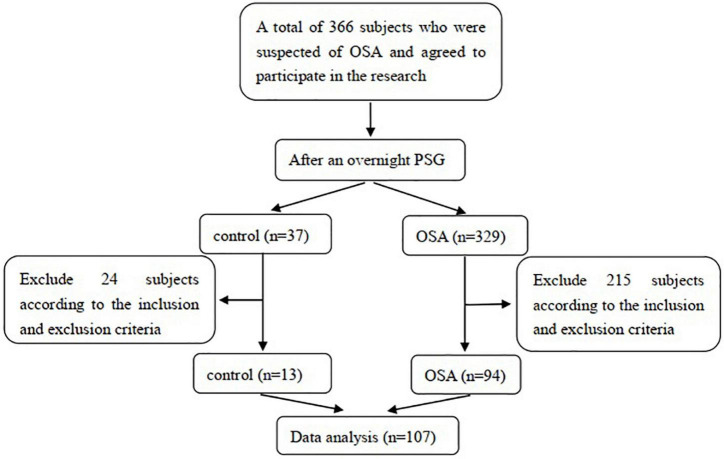
The process of subjects selection.

This cross-sectional study enrolled 94 OSA patients and 13 snoring patients who visited the Sleep Center of the Department of Respiratory and Critical Care Medicine, First Hospital of China Medical University, between January 2017 and August 2021. We estimated the sample size before the data analysis based on the results of the pre-experiment, and the target power of the design was 80%. The inclusion criteria were as follows: (1) patients with snoring symptoms suspected of OSA; (2) completion of overnight polysomnography (PSG) to confirm or rule out OSA; (3) aged 18–75 years old; and (4) agreement to participate in this study. The potential population was excluded if they (1) had diseases that influenced the level of sTREM2, such as previously diagnosed neurodegenerative diseases, mental illness; (2) had diseases that could induce cognitive impairment such as diabetes, hypertension, hyperlipidemia, and cardiovascular disease; (3) had conditions that affected nocturnal oxygen level or sleep structure, such as chronic airway disease, overlap syndrome, restless leg syndrome, insomnia, obesity hypoventilation syndrome, alcohol or sedating medicine intake; and (4) had previously or currently received OSA treatment. The enrolled subjects were divided into a control group or OSA group according to the PSG results. This study was approved by the Ethics Committee of the First Hospital of China Medical University. All subjects in this study received detailed information regarding the research and provided informed consent following the Declaration of Helsinki. The subject selection process is illustrated in Figure 1.

### Demographic Information Collection

The trained researchers collected basic information of the subjects before PSG monitoring on the night of monitoring, including sex, age, height, weight, smoking history, drinking history, past medical history, medication history, and major symptoms (including snoring, awakeness, daytime sleepiness, dry mouth, discomfort after awakening, and sexual dysfunction).

### Questionnaires

The participants completed questionnaires under the guidance of the researchers, including the Epworth Sleepiness Scale, PSQI, Mini-Mental State Examination (MMSE) scale, and Montreal Cognitive Assessment (MoCA) scale. All questionnaires were in Chinese.

### Polysomnography

All subjects underwent overnight PSG monitoring using a device (Alice 5 or SOMNO screen). The PSG monitor recorded the patients’ electroencephalogram, electrooculogram, electromyogram of the submental muscles, oral-nasal airflow, snoring, chest and abdominal wall movements, and peripheral arterial oxygen saturation. The technicians evaluated the data based on criteria established by the American Academy of Sleep Medicine (Berry et al., 2017). Apnea was defined as the peak signal excursion dropping by ≥90% of baseline levels for ≥10 s. Hypopnea was defined as a peak signal excursion dropping by ≥30% of baseline levels with ≥4% arterial oxygen desaturation for ≥10 s. Additionally, OSA was diagnosed when the apnea and hypopnea index (AHI) was ≥5 events/h.

### Blood Sample Collection and Serum sTREM2 Measurement

Fasting venous blood was collected in the morning after the end of the monitoring (6:30–7:00 am) and centrifuged (3,000 rpm, 10 min). Serum was collected and stored at −80°C. Serum sTREM2 was quantified by enzyme-linked immunosorbent assay (Abcam, Cambridge, United Kingdom) according to the manufacturer’s instructions, at the Respiratory Laboratory of the First Hospital of China Medical University.

### Statistical Analysis

Data were analyzed using SPSS 22.0. The normality of the data was tested using the Shapiro–Wilk test. Normally distributed continuous data are presented as the mean ± SD. For these data, comparisons between the two groups were evaluated using a *t*-test. Non-normally distributed continuous data are presented as median and interquartile range (IQR). For these data, the Mann–Whitney U-test was used to compare the two groups. The enumeration data were presented as percentages, and comparisons between groups were performed using the chi-square test. Covariance analysis was used to compare adjusted sTREM2 levels between the OSA and control groups. Univariate linear regression and stepwise multivariate linear regression were used to analyze the factors influencing sTREM2 levels in patients with OSA. A receiver operating characteristic (ROC) curve was used to assess the predictive value of serum sTREM2 for mild cognitive impairment (MCI) in patients with OSA. Differences were considered statistically significant at *p* < 0.05.

## Results

### Characteristics of Subjects

The characteristics of the enrolled patients were summarized in Table 1. Compared with the control group, the body mass index, sleepiness score, AHI, the time ratio of oxygen saturation below 90% (SIT90), longest apnea time, oxygen depletion index (ODI), percentage of N1 + N2 sleep stage, and micro-arousal index were higher in the OSA group, whereas the MoCA score, lowest oxygen saturation, mean oxygen saturation, sleep efficiency, and percentage of N3 sleep stage and rapid eye movement (REM) sleep stage were lower in the OSA group.

**TABLE 1:** Characteristics of enrolled subjects.

	Control group (*n* = 13)	OSA group (*n* = 94)
Age (years)	38(34−46)	46(33.75−54)
Sex (male,%)	61.54%	68.09%
Body mass index (kg/m^2^)	25.04 ± 4.04	27.92 ± 4.04^a^
Sleepiness score	5(3.50−8)	10(5−15)^a^
PSQI score	8(4−10.50)	6(4−10)
MMSE score	28(27−30)	28(26−29)
MoCA score	27(23.50−28)	22(24−26)^a^
AHI (events/h)	2.6(1.35−3.80)	34.20(19.68−58.15)^a^
Lowest oxygen saturation (%)	90(87−93)	80(71−84)^a^
Mean oxygen saturation (%)	96.5(96.05−97)	94(93−95.35)^a^
SIT90 (%)	0(0−0.19)	6.75(1.10−24.88)^a^
Longest apnea time (s)	15(0−27.40)	59.20(39.25−80.75)^a^
ODI (events/h)	2.33(0.60−6.24)	35.50(19.70−55.25)^a^
Sleep efficiency (%)	92.99(85.71−95.43)	82.29(75.36−93.97)^a^
N1 + N2 (%)	59.27 ± 4.97	71.92 ± 17.89^a^
N3 (%)	20.62(18.26−22.26)	14.03(8.21−27.40)^a^
REM (%)	18.72(17.90−22.31)	5.37(0.77−12.63)^a^
Micro-arousal index (events/h)	3.92(0.33−20.25)	28.25(11.55−45.80)^a^

*The normally distributed data were presented as mean ± SD, the comparison was evaluated by t-test; The non-normally distributed data were presented as the median (IQR), the comparison was evaluated by the Mann–Whitney U test; a, compared with control group, p < 0.05. AHI, apnea-hypopnea index; MMSE, Mini-Mental State Examination; MoCA, Montreal Cognitive Assessment; SIT90, the time ratio of oxygen saturation below 90%; ODI, oxygen depletion index; OSA, obstructive sleep apnea; PSQI, Pittsburgh Sleep Quality Index; REM, rapid eye movement.*

### sTREM2 Level

**FIGURE 2:**
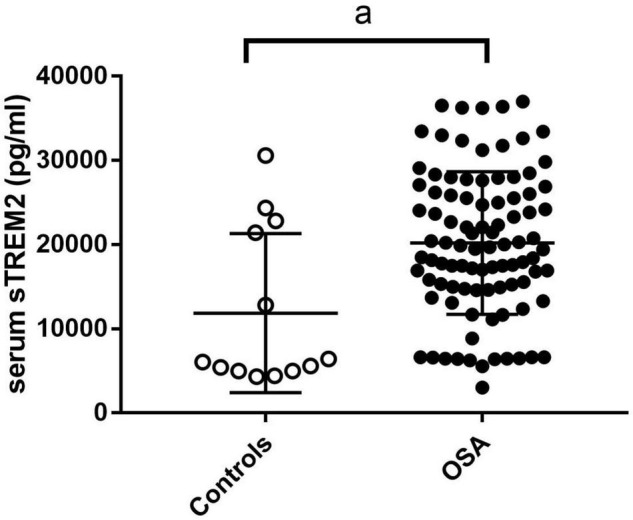
The sTREM2 level in control group and OSA group. The comparison was evaluated by covariance analysis; a, compared with the control group, *p* < 0.05. OSA, obstructive sleep apnea; sTREM2, soluble triggering receptor expressed on myeloid cells 2.

We compared sTREM2 levels between the control and OSA groups after adjusting body mass index. The results showed that the level of sTREM2 in the OSA group was higher than that in the control group (Figure 2).

### Influencing Factors of sTREM2 Level in OSA Patients

To further analyze the factors influencing sTREM2 levels in patients with OSA, we performed univariate linear regression on serum sTREM2 and various factors. The results showed that MoCA, AHI, ODI, and micro-arousal index were associated with the level of serum sTREM2 in OSA patients. The results are summarized in Table 2.

**TABLE 2:** The results of univariate linear regression analysis.

	*R*	*R* ^2^	β	*t*	*p*
MoCA	0.32	0.10	−0.32	−3.24	<0.01
AHI	0.37	0.14	0.37	3.78	<0.01
ODI	0.47	0.22	0.47	5.10	<0.01
Micro-arousal index	0.35	0.12	0.35	3.58	<0.01

*AHI, apnea-hypopnea index; MoCA, Montreal Cognitive Assessment; ODI, oxygen depletion index.*

Second, stepwise multivariate linear regression analysis was performed with the level of sTREM2 included as a dependent variable, and the level of MoCA score, AHI, ODI, and micro-arousal index were included as independent variables. The results showed that sTREM2 levels in OSA patients were affected by the MoCA score and ODI (Table 3). OSA patients with lower MoCA scores and higher ODI levels were more likely to have high serum sTEM2 levels. There was no multicollinearity among the factors.

**TABLE 3:** The results of multiple linear regression analysis.

	*R*	*R* ^2^		β	*t*	*p*	VIF
Step 1	0.47	0.22	constant	–	8.98	<0.01	–
			ODI	0.47	5.10	<0.01	1.00
Step 2	0.54	0.29	constant	–	4.97	<0.01	–
			ODI	0.44	4.93	<0.01	1.01
			MoCA	−0.27	−3.03	<0.01	1.01

*Step 1 excluded MoCA, AHI, and micro-arousal index; Step 2 excluded AHI, and micro-arousal index. MoCA, Montreal Cognitive Assessment; ODI, oxygen depletion index.*

### Predictive Value of sTREM2 for MCI of OSA Patients

**FIGURE 3:**
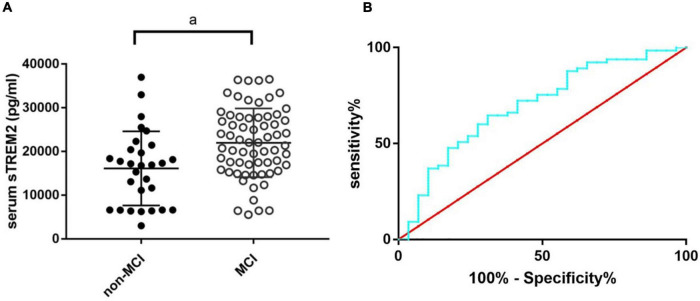
The sTREM2 level in OSA patients with and without MCI **(A)** and the ROC curve analysis of serum sTREM2 in discriminating OSA patients with MCI from those without **(B)**. The comparison was evaluated by *t*-test; a, compared with the control group, *p* < 0.05. MCI, mild cognitive impairment; OSA, obstructive sleep apnea; ROC, receiver operating characteristic; sTREM2, soluble triggering receptor expressed on myeloid cells 2.

The MoCA score is a useful tool with high sensitivity and specificity for MCI detection (Ciesielska et al., 2016). As the serum sTREM2 level in OSA patients was associated with the MoCA score, we further analyzed the value of serum sTREM2 to detect subjects with MCI among OSA patients based on the MoCA score (MoCA score range 17–26 was considered as MCI) (Nasreddine et al., 2005). The serum sTREM2 level was higher in OSA patients with MCI (Figure 3A, *p* < 0.01), and the receiver operating characteristic curve showed the area under the curve was 0.70 (95%CI: 0.58–0.81), the sensitivity was 64.62%, and the specificity was 68.97% (Figure 3B), with a cutoff value of serum sTREM2 >18,437 pg/ml. These results suggest that serum sTREM2 may have potential as a biomarker for MCI in patients with OSA.

## Discussion

It is a hot topic that sTREM2 may be a potential biomarker or therapeutic target for cognitive impairment caused by various diseases (Suárez-Calvet et al., 2016a,b; Ohara et al., 2019; Tanaka et al., 2019; Weber et al., 2020; Mo et al., 2021). However, little is known about the relationship between sTREM2 and OSA or OSA-related cognitive impairment. In this study, we compared the level of serum sTREM2 between OSA patients and snoring subjects and found that the level of serum sTREM2 was increased in OSA patients, and the increase in sTREM2 in OSA patients was associated with the MoCA score and ODI. In addition, ROC curve analysis showed that serum sTREM2 has the potential to be a biomarker for OSA-related MCI.

TREM2 is a transmembrane glycoprotein immune receptor that plays a vital role in the regulation of microglia-related immune responses and inflammatory cytokine production (Hamerman et al., 2006). Studies have found that deletion of TREM2 aggravates the neuroinflammatory response induced by lipopolysaccharide *in vitro* and *in vivo* (Zhang et al., 2020). sTREM2 is the soluble form of TREM2 derived from the proteolytic cleavage of the protein. The levels of sTREM2 in the CSF and blood are considered biomarkers for neurodegenerative diseases (Kim and Kim, 2020). In this study, we found that serum sTREM2 levels were higher in OSA patients than in snoring subjects. However, the underlying mechanisms remain unclear. Previous studies have found that increased levels of CSF or plasma sTREM2 in neuroinflammatory diseases are related to the level of CSF or peripheral inflammatory factors (Bekris et al., 2018; Rauchmann et al., 2020; Mo et al., 2021). The levels of inflammatory factors in OSA patients are increased because of the oxidative stress caused by intermittent hypoxia and sleep fragmentation (Atrooz and Salim, 2019; Liu et al., 2020). Elevated inflammatory cytokines induce the expression of interferon-induced transmembrane protein 3, which binds to γ-secretase and upregulates its activity (Hur et al., 2020), thereby increasing the cleavage of sTREM2. Therefore, we suspect that the inflammatory condition increases sTREM2 levels in OSA patients. Moreover, regression analysis showed that serum sTREM2 levels were associated with ODI. This further confirmed that the inflammatory response may be the cause of the changes in sTREM2 in OSA patients, because ODI is related to inflammatory factors (Svensson et al., 2012). However, further studies are required to explore the underlining mechanisms.

In addition to the ODI, we found that the MoCA score was associated with the level of serum sTREM2 in OSA patients, whereas the MMSE score was not. However, Tanaka et al. (2019) found that plasma sTREM2 was associated with MMSE score in non-obese diabetes patients. This may be because the MoCA score assesses a broader range of cognitive domains (Chen et al., 2011), cognitive impairment in OSA patients mainly manifests as MCI, and the MoCA test is better for the detection of MCI than MMSE (Ciesielska et al., 2016). Moreover, the area under the ROC curve was 0.7 for sTREM2, differentiating OSA patients with MCI from those without. This indicates that serum sTREM2 has the potential to be a biomarker for MCI in OSA patients. These results were similar to previous studies (Suárez-Calvet et al., 2016a,b; Bekris et al., 2018; Ohara et al., 2019; Tanaka et al., 2019; Weber et al., 2020; Mo et al., 2021), although most tested sTREM2 levels in the CSF. In dementia and MCI, microglia in the central nervous system are activated and expression of TREM2 increases, with release of sTREM2 into the CSF. It has been reported that sTREM2 can across the blood-brain barrier, and the level of sTREM2 in plasma is related to that in the CSF (Bekris et al., 2018). Because of the increased permeability of the blood-brain barrier in OSA patients, the sTREM2 in serum can be derived from the CSF (Zolotoff et al., 2020). However, since monocyte in the blood can also express TREM2 and release its soluble form (Klesney-Tait et al., 2006), the source of serum sTREM2 in OSA patients is unclear. Further studies are required to confirm this hypothesis.

Overall, the underlining mechanisms of sTREM2 and cognitive impairment remain unclear. As we all know, TREM2 has an important effect on maintaining the neuroprotective function of the microglia. TREM2 interacts with one of its ligands, and the signal is propagated via the adaptor proteins DNAX activation protein 10 (DAP10) and DAP12, resulting in the activation of phosphatidylinositol 3-kinase, spleen tyrosine kinase, mammalian target of rapamycin, and mitogen-activated protein kinase, respectively (Peng et al., 2010). The activation of these proteins mediates the effects of microglial phagocytosis, anti-inflammation, and cellular metabolism (Yeh et al., 2016; Wang et al., 2020; Zhang et al., 2020), and then plays a neuroprotective role. Under oxidative stress, cleavage of TREM2 caused by neuroinflammation creates sTREM2 and stops the TREM2 signaling cascade, leading to cognitive impairment. Therefore, increased sTREM2 levels seem to be a consequence rather than a cause of cognitive impairment. However, sTREM2 levels may change dynamically in different stages of Alzheimer’s disease. Are there similar alterations in OSA? Is there an association between sTREM2 and other biomarkers of neurodamage? All these questions demand deep exploration.

Sleep fragmentation is a critical characteristic of OSA. Undoubtedly, we found that sleep efficiency and the proportion of REM were decreased, and the proportion of light sleep and arousal index were higher in OSA patients than in snoring subjects. Recent studies have confirmed that sleep plays an important role in regulating TREM2-associated microglial activity (Bellesi et al., 2017; Hu et al., 2021). Hu et al. found that CSF sTREM2 in cognitively normal older adults was positively correlated with the sleep efficiency score assessed by the PSQI, and this relationship was more obvious in amyloid-positive adults (Hu et al., 2021). Another study suggested that sTREM2 in the CSF has a significant diagnostic value for sleep disorders in Parkinson’s disease (Mo et al., 2021). Therefore, is there a relationship between elevated serum sTREM2 levels and sleep? Univariate linear regression showed that serum sTREM2 level was associated with the micro-arousal index. The micro-arousal index is considered the gold standard for sleep fragmentation (Bonnet et al., 2007), and has an important impact on cognitive impairment (Xu et al., 2020). However, multivariate linear regression analysis showed that there was no association between micro-arousal and serum sTREM2 levels in OSA patients. The difference may have resulted from the different subjects and sources of sTREM2 in our study. Since almost all OSA patients, especially those with severe OSA, suffer from disturbances of sleep structure, the difference in sleep parameters between the groups in our study was not obvious. Therefore, further large-scale longitudinal studies and animal mechanistic research are required.

Our study has some limitations. First, we did not assess TREM2 gene mutation conditions among the enrolled patients. This could have influenced the results because the level of sTREM2 is associated with the mutation status (Piccio et al., 2016). Second, we performed ROC curve analysis to assess the predictive value of serum sTREM2 by taking the results of the MoCA score rather than the pathology results as the standard of MCI diagnosis, which might limit the generalization of the findings. Finally, due to the cross-sectional design, it is difficult to conclude a causal relationship between serum sTREM2 levels and OSA-related MCI. Large-sale longitudinal and mechanistic studies are required to elucidate the role of serum sTREM2 in OSA and its related cognitive impairment.

In conclusion, serum sTREM2 levels were higher in patients with OSA. Patients with a low MoCA score and high ODI were more likely to have a high level of serum sTREM2. sTREM2 has the potential to be a biomarker of MCI in patients with OSA. These findings suggesta TREM2-associated pathophysiological process in OSA-related cognitive impairments. Further basic and clinical research is needed to validate the role of TREM2 and sTREM2 in OSA.

## Data Availability Statement

The raw data supporting the conclusions of this article will be made available by the authors, without undue reservation.

## Ethics Statement

The studies involving human participants were reviewed and approved by the ethics committee of the First Hospital of China Medical University. The patients/participants provided their written informed consent to participate in this study.

## Author Contributions

XJ contributed to study design, data collection and analysis, manuscript drafting, and final approval of the version to be published. ZY and JH were in charge of the process of data collection, enzyme-linked immunosorbent assay, critical manuscript revision, and final approval of the version to be published. WZ, GS, DC, and FL were in charge of the process of data collection and analysis and final approval of the version to be published. LF provided language help, writing assistance, critical manuscript revision, and final approval of the version to be published. WW contributed to study design, data interpretation, critical manuscript revision, and final approval of the version to be published. All authors contributed to the article and approved the submitted version.

## Conflict of Interest

The authors declare that the research was conducted in the absence of any commercial or financial relationships that could be construed as a potential conflict of interest.

## Publisher’s Note

All claims expressed in this article are solely those of the authors and do not necessarily represent those of their affiliated organizations, or those of the publisher, the editors and the reviewers. Any product that may be evaluated in this article, or claim that may be made by its manufacturer, is not guaranteed or endorsed by the publisher.
